# Cryogenic spray quenching of simulated propellant tank wall using coating and flow pulsing in microgravity

**DOI:** 10.1038/s41526-022-00192-w

**Published:** 2022-04-01

**Authors:** J. N. Chung, Jun Dong, Hao Wang, S. R. Darr, J. W. Hartwig

**Affiliations:** 1 Cryogenics Heat Transfer Laboratory Department of Mechanical and Aerospace Engineering, University of Florida, Gainesville, FL 32611-6300 USA; 2 NASA Glenn Research Center, Cleveland, OH 44135 USA

**Keywords:** Aerospace engineering, Mechanical engineering

## Abstract

In-space cryogenic propulsion will play a vital role in NASA’s return to the Moon mission and future mission to Mars. The enabling of in-space cryogenic engines and cryogenic fuel depots for these future manned and robotic space exploration missions begins with the technology development of advanced cryogenic thermal-fluid management systems for the propellant transfer lines and storage system. Before single-phase liquid can flow to the engine or spacecraft receiver tank, the connecting transfer line and storage tank must first be chilled down to cryogenic temperatures. The most direct and simplest method to quench the line and the tank is to use the cold propellant itself that results in the requirement of minimizing propellant consumption during chilldown. In view of the needs stated above, a highly efficient thermal-fluid management technology must be developed to consume the minimum amount of cryogen during chilldown of a transfer line and a storage tank. In this paper, we suggest the use of the cryogenic spray for storage tank chilldown. We have successfully demonstrated its feasibility and high efficiency in a simulated space microgravity condition. In order to maximize the storage tank chilldown efficiency for the least amount of cryogen consumption, the technology adopted included cryogenic spray cooling, Teflon thin-film coating of the simulated tank surface, and spray flow pulsing. The completed flight experiments successfully demonstrated that spray cooling is the most efficient cooling method for the tank chilldown in microgravity. In microgravity, Teflon coating alone can improve the efficiency up to 72% and the efficiency can be improved up to 59% by flow pulsing alone. However, Teflon coating together with flow pulsing was found to substantially enhance the chilldown efficiency in microgravity for up to 113%.

## Introduction

In NASA’s return to the Moon mission and future mission to Mars, a highly efficient cryogenic thermal-fluid management technology is among the indispensable requirements for successful lunar and mars space missions. The planned propellant fuel depot deployed in the Lower-Earth-Orbit (LEO) for future deep-space missions, and the human-carrying spacecraft flying lunar and mars missions are designed to utilize liquid cryogenic fuels and oxydizers^1–4^. For the human mars surface mission, one of the enabling technologies is the efficient transfer of propellant from the fuel depot to the spacecraft propellant storage tank^1^. The actual tank-to-tank propellant transfer, however, has yet to take place, mainly due to the lack of cryogenic quenching data in reduced microgravity^4^ for designing the transfer system. As the existing technology on cryogenic chilldown can only offer relatively very low efficiencies^5^ and it has not been developed under the microgravity conditions, a new technology with much higher efficiencies and verified under microgravity conditions is therefore needed for future space missions.

In order to maximize the storage tank chilldown efficiency, the technology proposed includes cryogenic spray cooling, Teflon thin-film coating of the tank inner surface, and spray flow pulsing. The completed flight experiments successfully demonstrated that cryogenic spray cooling is the most efficient cooling method for the tank wall chilldown in microgravity. Teflon coating together with flow pulsing was found to substantially enhance the chilldown efficiency in microgravity. The feasibilities of charge-hold-vent for tank chilldown and no-vent-fill for tank filling in microgravity were also successfully demonstrated.

According to publications by the members of the Space Cryogenic Thermal Management Group at NASA Glenn Research Center, Doherty et al.^6^ and Myer et al.^7^ provided the main areas of research and development for space cryogenic thermal management. The tank-to-tank transfer of propellants in space is composed of transfer line chilldown, receiver tank chilldown, and no-vent fill of the receiver tank. Among all three areas, receiver tank chilldown is considered the most important area as the amount of cryogen consumed is the largest. In this paper, we report an advance in microgravity tank wall chilldown heat transfer using a thin-film coating and spray cooling.

**Fig. 1:**
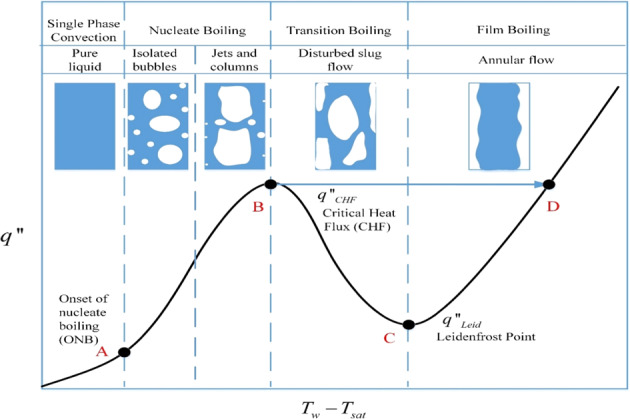
A typical boiling curve. This cure illustrates different boiling regimes and corresponding flow patterns.

The chilldown of the receiver tank wall by spray cooling using liquid cryogen is a liquid-to-vapor phase change quenching process that is characterized by the so-called “boiling curve” as shown in Fig. 1. This curve^8^ represents the tank wall surface heat flux,$q^{\prime\prime}$, plotted against the wall surface degree of superheating, $T_W - T_{{\mathrm{sat}}}$, where $T_W$ is the surface temperature and $T_{{\mathrm{sat}}}$ is the saturation temperature corresponding to the boiling fluid bulk pressure. A quenching process follows the route D→C→B→A. Therefore, during chilldown the heat transfer on the tank wall surface always experiences film boiling first due to a very hot tank wall surface. Because the heat fluxes in film boiling are relatively quite low, film boiling regime always occupies the major portion of the total quenching time. Accordingly, the thermal energy efficiency in the traditional quenching process is extremely low. According to Shaeffer et al.^5^, the average quenching efficiency is about 8% that provides a strong incentive to find more efficient methods for the space storage tank chilldown process.

Progressive advances in high power density electronics and high-performance energy systems have precipitated the need for innovative thermal management technologies to ensure reliable performance and reduce the payload of thermal management systems. Such systems include high current density propulsion systems, high power electronics for energy conversion, high power optical sensors, as well as high power microelectronics packaged within environmental enclosures. In order to manage the progressively increasing heat flux requirements for thermal management systems, a spray cooling system has been proposed and under constant development for the past sixty years. Liang and Mudawar^9^ indicated that spray cooling possesses several advantages: high flux heat dissipation, low and fairly uniform surface temperature, and ability to cool relatively large surface areas using a single nozzle.

In very recent papers, Liang and Mudawar^9,10^ provided a highly comprehensive, thorough, and complete review of the spray cooling research up to 2017. Almost all of the published papers were using water and refrigerants and we found only three papers on the study of terrestrial cryogenic spray cooling. Sehmbey et al.^11^ performed a liquid nitrogen spray cooling experiment to gather heat transfer characteristics to facilitate the operation of power electronics at very low temperatures. Four different nozzles at various pressures were used to study the variation in spray cooling heat transfer at liquid nitrogen temperature. The effect of nozzle and flow rate on the critical heat flux (CHF) and overall heat transfer characteristics were presented. Cooling heat fluxes close to 1.7 × 10^6^ W/m^2^ were realized at temperatures below 100 K. The mass flow rate range was from 6.1 × 10^4^ to 3.2 × 10^5^ kg/h m^2^. They demonstrated that a high heat flux (over 1.0 × 10^6^ W/m^2^) cooling technique, such as spray cooling, will have to be used to realize all the advantages of low-temperature operation. Following their experimental study, Sehmbey et al.^12^ further provided empirical correlations for liquid nitrogen spray cooling. They offered a general semiempirical correlation (based on macrolayer dryout model) for spray cooling CHF for different liquids and spray conditions. An empirical correlation for heat flux was also presented. They also pointed out the importance of surface roughness for spray cooling with liquid nitrogen. It was discovered that the rougher surfaces have significantly higher heat transfer rates and similar CHFs occurring at lower temperatures.

Somasundara and Tay^13^ investigated the intermittent liquid nitrogen spray cooling for applications, which require higher heater operating temperatures (−180 to 20 °C). This intermittent spray cooling process can be adjusted using the mass flow rate, pulsing frequency, and duty cycle (percentage of open time in one cycle) to match the required cooling rate on the target. The intermittent spray experiments were conducted for various ranges of surface temperatures.

Kato et al.^14^ studied the gravity effects on liquid spray cooling using a nickel-plated copper block in terrestrial and variable gravity conditions onboard parabolic flight. The copper block was first heated by seven cartridge heaters to a prescribed temperature and then cooled down by spraying water or CFC-113 onto the nickel-plated surface which is only 19 mm in diameter. They observed that the heat transfer in the low heat flux regime below the CHF was enhanced by the reduction in gravity for both fluids. However, the effects of reduced gravity act differently on these two fluids at CHF. The CHF for CFC-113 was decreased in a low gravity level whereas the CHF of water increased. Kato et al.^14^ also reported the vanishing of the gravity effect on the heat transfer at high spray volume mass fluxes.

As a follow-up study, Yoshida et al.^15^ conducted a more comprehensive study of the effects of gravity on spray cooling. Two different heaters were used in this study. One is similar to the copper block described by Kato et al.^14^ except that the surface was plated with chromium and 50 mm in diameter. The other was a glass prism plated with a thin transparent indium tin oxide (ITO) film as the heating element. This transparent glass heater was used in order to visualize the liquid deposition on the heater surface for steady-state spray cooling while a copper block was used for transient spray cooling test. The working fluids used were water and FC-72. A series of ground-based tests and parabolic flight tests were performed by varying the test parameters such as working fluid, heater surface orientation, heat flux at heater surface, the mass flux of the coolant as well as heater types. They reported that gravity level had little effect in the nucleate boiling regime. Moreover, they suggested a coupled effect of gravity and spray volume mass flux on CHF. In the case of a low spray volume flux, neither the magnitude nor the direction of gravity affected CHF. However, the CHF under reduced gravity is higher than that under the hypergravity by 10 percent. They also noted the significant influence of gravity on the film boiling regime when the Webber number was low. And they argued that the deterioration of the heat transfer during the film boiling in the case of low Webber number and reduced gravity condition is due to a lack of secondary impingement on the heater surface.

As indicated by the literature review above, we only found a handful of terrestrial cryogenic spray cooling research papers. However, there has been no attempt on microgravity and reduced gravity cryogenic spray cooling using either room-temperature liquids or cryogens. We believe that the current paper is the first to report the experimental data on cryogenic spray cooling in reduced gravity.

According to transient conduction heat transfer theory^16^, if two materials A and B were put together in contact, then the instantaneous heat flux $q^{\prime\prime}_{A \to B}$ from material A to material B is given by Eq. (1) below,

1 $$q^{\prime\prime}_{A \to B} = \frac{{k_A\left( {T_{A,i} - T_s} \right)}}{{\left( {\pi \alpha _At} \right)^{1/2}}} = - \frac{{k_B\left( {T_{B,i} - T_s} \right)}}{{\left( {\pi \alpha _Bt} \right)^{1/2}}}$$

Where $T_{A,i}\,and\,T_{B,i}$ are constant temperatures of A and B just before contact, respectively. Also, thermal conductivities of A and B are $k_A\,and\,\,k_B$, respectively. $\alpha _A\,and\,\alpha _{B}$ are thermal diffusivities of A and B, respectively. $T_s\,\,$ is the interface temperature, while t is the elapsed time after the contact.

We can see that $q^{\prime\prime}_{A \to B}$ is a function of $t^{ - 1/2}$ during the transient^17^. In essence, initially, the heat transfer rate between the two materials is very high, but it also drops off quickly. As a result, for the pulsed flow quenching process, at the moment when the pulsed flow is switched on in a duty cycle, the disk surface gets in contact with a fresh cooling fluid that induces a peak in the heat transfer rate that produces higher cooling rates than the streaming flow case. Based on Eq. (1), the duty cycle (DC) of the pulse flow is the dominant factor on the cooling enhancement solely by the exponential decay of the thermal transport in time, the effect of different periods is only of the second-order effect. Chung et al.^17^ found that the pulsed flow would raise the chilldown efficiency up to 67% over the continuous flow case for the convective chilldown of a metal pipe. Chung et al.^17^ also reported that the chilldown efficiency increases with decreasing DC, but it is insensitive to the period.

The basis of quenching heat transfer enhancement by the low-thermal conductivity coating is given in Chung et al.^18^. As shown in Chung et al.^19^ for convective metal tube chilldown, both thermal diffusivities and thermal conductivities ratios between the stainless steel and the coating material are involved, but clearly, the thermal conductivity ratio dominates the transient process such that the low-thermal conductivity coating can facilitate more than an order of magnitude larger drop of the tube wall surface temperature for the initial period after the quenching is initiated. Chung et al.^18,19^ used thin-layers of Teflon for enhancing heat transfer during chilldown of a metal pipe and they found that the coatings could increase the chilldown heat transfer efficiency up to 109 and 176% in terrestrial gravity^19^ and microgravity^18^, respectively.

The primary objective of the current set of microgravity experiments is to obtain transient quenching heat transfer characteristics of a typical storage tank wall surface simulated by a metal circular disk. The disk transient temperature history or a chilldown curve during spray quenching from room temperature to LN2 saturation temperatures was measured. One of the two disks was coated with a low-thermal conductivity thin Teflon layer to evaluate the heat transfer enhancement. The effectiveness of the coating was evaluated by a comparison of chilldown efficiencies with a coating to those of a bare surface disk. Tests were carried out with a set of pulse flow conditions that includes 40, 50, and 70% duty cycles with 1 s period over a wide range of test section inlet pressure levels and corresponding mass flow rates. The effectiveness of the pulse flow was evaluated by a comparison of chilldown curves with flow pulsations to those with constant and continuous flows.

## Methods

### Experimental system

**Fig. 2:**
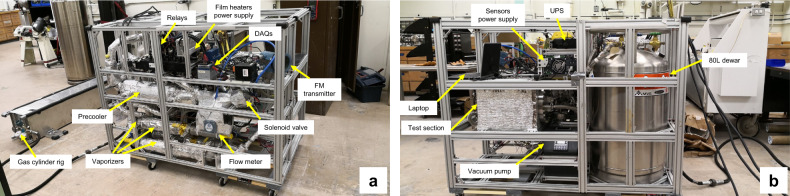
Photographic images of the experimental system. **a** Front view, **b** Back view.

Figure 2 shows the photos of the parabolic flight experimental system. The test chamber of this apparatus is designed to comprise two nozzles to spray liquid nitrogen onto two separate stainless-steel circular disks simultaneously. All the system components except the high-pressure gas cylinder fit into a (L × W × H) 1.4 m × 0.8 m × 1 m 8020 aluminum frame. This highly integrated thermal-fluid system was installed on the floor of ZERO-G Corporation’s Boeing 727-200 F aircraft^20^ to perform the parabolic flight disk chilldown experiment in a simulated reduced gravity environment. The reduced gravity is achieved through flying the aircraft in a parabolic trajectory and each parabola provides about 17–20 s reduced gravity (10^−2^g) period.

**Fig. 3:**
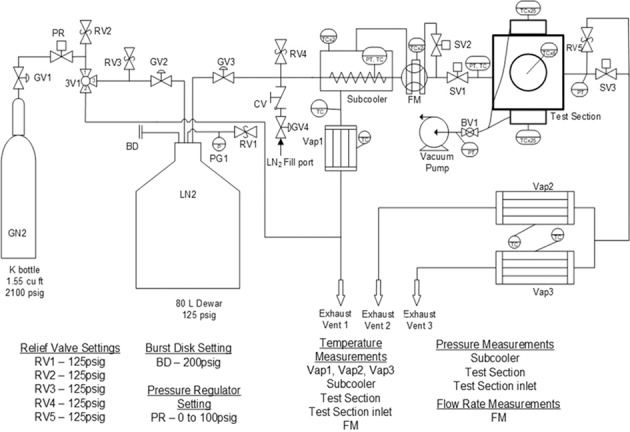
The fluid piping schematic and instrumentation diagram of the experimental system. The valves and important components of the fluid network. Relief valve settings, the burst disk setting, and pressure regulator settings are also included. BD burst disk, BV ball valve, CV check valve, FM flow meter, GN2 gaseous nitrogen, GV globe valve, LN2 liquid nitrogen, PG pressure gauge, PR pressure regulator, PT pressure transducer, RV relief valve, SV solenoid valve, TC thermocouple, Vap vaporizer, 3 V three-way valve.

The experimental apparatus consists of four essential fluid units together with auxiliary components, fluid piping and instrumentations. The fluid piping schematic and instrumentation diagram is shown in Fig. 3. The 80 L double-wall cryogenic dewar supplies the LN2 to the test section for performing the disk chilldown test as well as provides the LN2 for the prechilling of all the fluid components upstream of the test section prior to the actual chilldown test. Before the experiment, the 80 L dewar is topped off with industrial-grade liquid nitrogen from a standard Airgas 180-Liter dewar through the LN2 fill port. The subcooler is essentially a simple shell-tube heat exchanger and it serves two functions. The first one is to subcool the liquid nitrogen before it enters the test section such that the thermodynamic state of the liquid entering the test section can be determined. During the subcooler operation, the inner finned tube of the subcooler is totally submerged in the liquid nitrogen bath on the shell-side. Since the pressure inside the tube is always higher than that on the shell-side, the liquid nitrogen bath is always colder than that inside the tube. Thus, heat is removed from the liquid in the tube side. The second function is to save the liquid nitrogen during the pre-test chilldown of the upstream components of the test section. The vapor generated on the shell-side is separated by gravity and is vented outside the system.

**Fig. 4:**
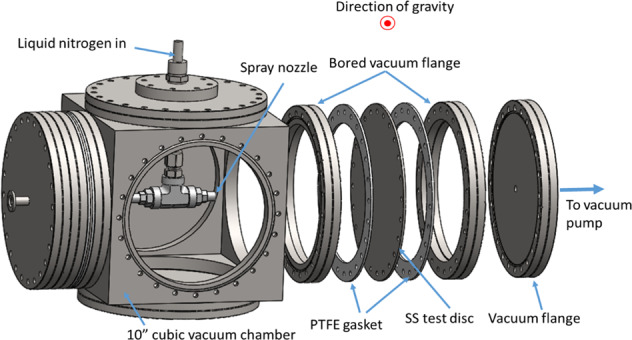
A CAD drawing of the test section. The test chamber is 10-inch cubic and it houses two spray nozzles.

**Fig. 5:**
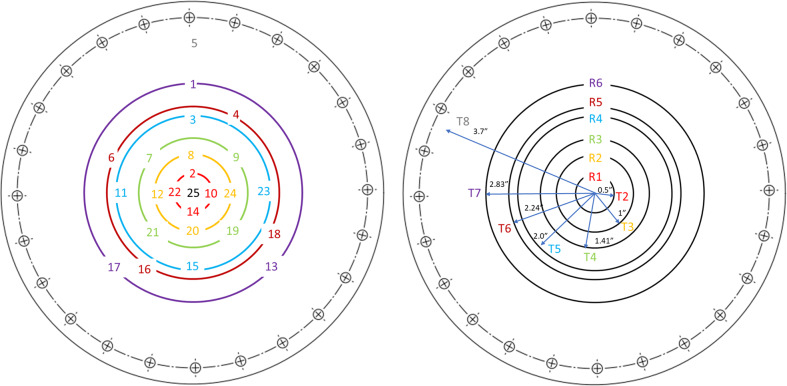
Schematic of thermal couple placement. Locations of 25 thermal couples are shown on six concentric circles.

The test section is basically a vacuum chamber where the cryogenic spray cooling of the disk takes place during the experiment and it is made mostly by off-the-shelf vacuum components. The exploded view of the test section is given in Fig. 4, which shows the configuration of the two test disks and two spray nozzles. Two spray nozzles are placed at the center between two disks inside the 10-inch cubic vacuum chamber. It is noted that the flow direction is perpendicular to the disk and the flow is parallel to the gravity. Two stainless-steel disks, cut from a 16-gauge 304 stainless-steel sheet with a thickness of 0.058 inches, were mounted at opposite sides of the chamber, for example, the front and back or top and bottom. Depending on the purpose of the test, either one set of nozzle and plate or two sets of nozzles and plates are installed. For the ground test, only a single nozzle and one plate were installed inside the test chamber. The orientation of the heat transfer disk surface with respect to the gravity direction is varied by placing the stainless-steel disk at the bottom, side, or top of the cubic test chamber, and they are referred to as upward, vertical, and downward configurations. For the flight tests, two different disk plates and two nozzles were installed as shown in Fig. 4. Each disk is sandwiched by two bored vacuum flanges. Two PTFE gaskets were compressed against the stainless-steel disk such that the cubic test chamber and the back of the disk can be sealed. The outermost flange on each test disk assembly is connected to the vacuum pump such that the back of the stainless-steel disk is insulated from the surroundings by drawing a vacuum to minimize the parasitic heat input from the outside environment. For measuring the disk transient temperature history during the chilldown, 25 thermocouples (TCs) were soldered to the vacuum side (back) of each stainless-steel disc. As shown in Fig. 5, a total of 24 TCs were distributed on the 6 concentric circles (6 rings, R1, R2, …, R6) in addition to one (TC25) placed at the center. Only one TC (TC5) is placed on the outermost point near the outer boundary. TC5 is 3.7″ away from the center of the plate. Eight MINCO film heaters (Hap 6945 and 6946) are attached to the back of the disk to reheat the stainless-steel plate back to the initial temperature for the next test after each chilldown test.

The flow coming out of the test section goes into two separate vaporizers in parallel (labeled as Vap2 and Vap3 in Fig. 3). The vaporizers are basically heat exchangers made from tube bundles, which evaporate any remaining liquid nitrogen coming out of the test section and also heat up the cold nitrogen vapor to above 0 °C before venting pure vapor out of the system. Each vaporizer is made by packing eight grooved copper tubes that have star-shaped inner insertions into a 2.5″ schedule 40 stainless-steel pipe. The tube bundles are heated up to 200 °C before each test by a high-temperature heating tap that is wrapped around the outer surface of the stainless-steel pipe. The Labview program monitors and controls the on and off of the heating tap by the combination of a K-type thermocouple, NI 9211 thermocouple input module, NI 9472 digital output module, and a mechanical relay. If the experiment is performed onboard the aircraft, the gas coming out of the vaporizers is vented outside the aircraft cabin through rubber hoses that connect to the vent ports on the cabin wall. Similarly, another vaporizer, Vap1 ensures the proper venting of gaseous nitrogen coming out of the subcooler.

The data acquisition system including the Labview program and National Instrument Compact DAQ hardware collects all sensor data and displays the real time on a laptop at a sampling rate of 16 Hz. NI 9214 TC modules read all the T-type TCs (Omega). NI 9205, an analog input module, reads all the voltage signals from pressure transducers (Omega PX 409V5A) and the 4–20 mA current signals (through a 249-ohm resistor) from the Coriolis liquid flow meter (Micro motion CMF025). The Labview program controls the opening and closing of the solenoidal valve, SV1, through a combination of NI USB 6009 and a Solid-State relay. In the case of a continuous spray, the relay energizes the solenoid valve after receiving a continuous voltage signal. However, in the case of an intermittent spray, the relay energizes and de-energizes SV1 according to a rectangular waveform voltage signal from the VI.

In the current experiment, we used two types of disks. In addition to the bare surface stainless-steel disk, we also added a coated disk that is a stainless-steel disk coated with a low-thermal conductivity thin-film Teflon layer on one side of the disk surface. Specifically, the coating material was made of Fluorinated Ethylene Propylene (FEP) by DuPont and classified by DuPont as Teflon 959G-203 that is a black color paint and has a thermal conductivity of 0.195 W/mK (DuPont publication^21^). The coating was put on using the dip and drain process. The thickness of the coating is estimated at around 20–30 microns. The thickness of the Teflon coating was estimated by previous experiences obtained from an identical coating method. In our previous pipe chilldown experiment^19^, the thickness of the coating on the tube inner surface was measured by the high-resolution X-ray computer tomography (CT) scan. Since we used the same method to coat the disk plate as that used in the tube and expected the thickness of the coating is similar to that of the tube coating.

### Experimental procedure

To perform the chilldown test, mainly four steps are followed, and these are initial starting, precooling, testing, and reheating. The initial starting is the step where all the electrical devices are turned on. This includes running the preprogrammed Labview script and turning on the vacuum pump (Turbo Lab 80). Once the Labview script is running, it will automatically set the output voltage of the DC power supply for the pressure transducers and turn on the heating cables of the vaporizers. This step takes about 30 min mainly due to the time required for heating up the vaporizers to 200 °C. Meanwhile, the globe valves, GV1, GV2, GV3 are open for the next step. The second step involves the precooling and prechilling of all the piping and fluid components upstream of the test section to make sure that only the liquid phase of working fluid enters the test section and it proceeds first by rotating the three-way valve, 3V1, from GV1 to GV2, and setting the desired testing pressure on the pressure regulator, PR. Once the solenoidal valve SV2 is open by clicking the virtual button on the Laptop screen, then the liquid nitrogen starts to flow from the 80 L dewar into the shell-side of the subcooler. Before the liquid nitrogen can fill up the shell-side of the subcooler, the flow path upstream of the test section has to be chilled down. This step prevents the boil-off of liquid nitrogen before it flows into the test section. Once the inner tube of the subcooler is full (can be determined by the profile reading of the TC located inside the subcooler), the system is ready for the experiment. The chilldown test is started simply by clicking the virtual start bottom on the laptop screen, then the solenoid valve, SV1, will be opened according to the preset waveform signals either to continuously or intermittently flow nitrogen into the test section for spraying on the target disks. Once all the temperature readings from the TCs drop to the liquid nitrogen temperature and maintain at a steady-state, the disk chilldown experiment is complete. The solenoid valve, SV1, is then closed by clicking the virtual stop bottom. Next, the reheating step starts to prepare the stainless-steel disc plates for the next test. To heat up the plates after chilldown, the film heaters are turned on by clicking the heating virtual bottom on the screen. Once the plates are heated back to room temperature, the film heaters are turned off. This marks the beginning of the next cycle of testing which starts with the precooling step.

In addition to the experimental procedure discussed above, next, we need to mention the simulated microgravity environment on the parabolic flight. The variable gravity condition inside the airplane was created when the airplane is flying a parabolic trajectory^20^. The microgravity period is always sandwiched between two 1.8-g periods where g is the earth’s gravity of 9.81 m/s^2^. The microgravity period nominally lasts between 18-25 s. For our research flights, in order to maintain the acceleration levels within ±0.01 g, the microgravity period is around 18–20 s.

### Experimental uncertainty

Table 1 lists all the uncertainties or the independently measured quantities. The uncertainty for the chilldown thermal efficiency is discussed above in the Results section.

**Table 1:** Measured quantities and their uncertainties.

Symbol	Quantity	Measurement method	Uncertainty
T	Plate temperature	T-type thermocouple	1 K or 1.5% below 273 K
P_c_	Test section pressure	Kulite CTL-190M140BARA	7 kPa
δ	SS plate thickness	Calipers	0.03 mm
R_x_	Radial position of TCs on Ring x	Ruler	1.6 mm (1/16″)
ṁ	LN_2_ mass flow rate	Coriolis flow meter	0.3%
M	Mass of the fluid components (tube, tee, nozzle)	scale	0.1 g

### Reporting summary

Further information on research design is available in the Nature Research Reporting Summary linked to this article.

## Results

### Microgravity experiments completed

The University of Florida flight team led by Professor Jacob Chung performed parabolic flight experiments in one flight on 12 November 2018. Since we only had one parabolic flight to perform the experiments, we were only able to use one test section configuration. As shown in Fig. 4, the test section is composed of two disks facing each other and they were sprayed on by two separate spray nozzles, respectively, sharing the same liquid nitrogen feed line such that we could obtain two sets of chilldown data with identical spray flow condition simultaneously in one experimental run. As discussed in the Method section, one target was a stainless-steel disk coated with a low-thermal conductivity Teflon 959G-203 thin-film layer^21^, and the other was the bare surface stainless steel disk without any coating to serve as the baseline case for evaluating the coating effects. Table 2 lists the six cases we performed during the one parabolic flight experiment. Due to the limited runs allowed, only three pulse flow cases were performed. Also, the only one-second period was chosen due to limited microgravity times and the relative insensitivity of period to heat transfer. It is noted that the gravitational acceleration for Mars is 3.711 m/s^2^ that is 37.8% of earth’s gravity of 9.81 m/s^2^. In our notation, the Martian gravity is 0.378 g where g is earth gravity. As mentioned above, the gravity under simulated microgravity environment in the parabolic flight is about ±0.01 g.

**Table 2:** Experimental conditions for the six flight cases.

Case	P_in_ (psig)	Duty cycle (%)	Period (second)	g-level
1	80	100	NA	microgravity
2	80	40	1	microgravity
3	80	70	1	microgravity
4	60	100	NA	microgravity
5	90	100	NA	microgravity
6	90	50	1	microgravity

### Typical chilldown curves

As shown in Fig. 5, for measuring the disk transient temperature variation history at different locations during the chilldown for determining the quenching efficiency, 25 thermocouples (TCs) were soldered to the vacuum side (back) of the test disk. A total of 23 TCs are distributed on six concentric circles (six rings, R1, R2, …., R6) in addition to one (TC25) placed at the center and one (TC5) placed on the outside near the outer boundary. TC5 is 3.7″ away from the center of the disk plate

**Fig. 6:**
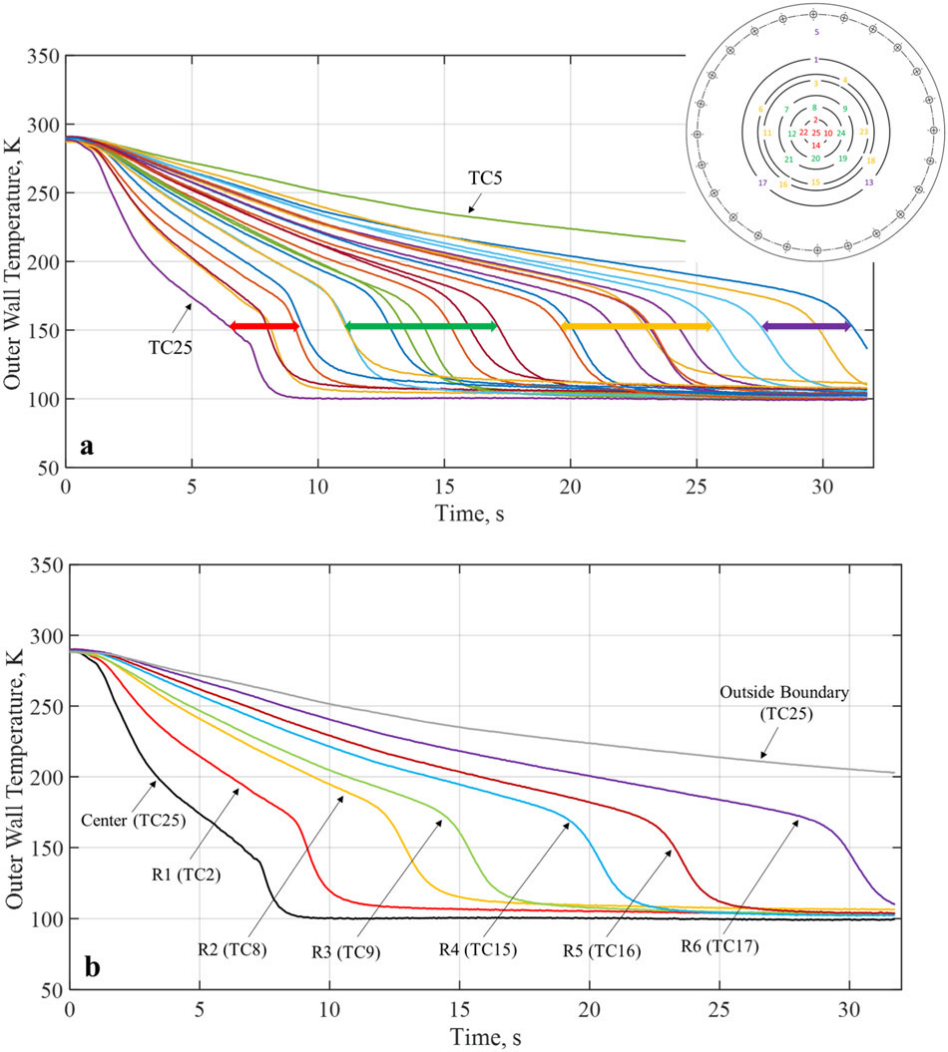
Typical chilldown curves. **a** A typical set of full 25 chilldown curves from a bare surface stainless-steel disk spray chilldown experiment, **b** a simplified set of chilldown curves from Fig. 3a.

A chilldown curve for a local point on the disk is defined as the transient temperature history at this point recorded by a thermal couple (TC) during the chilldown process. So, these curves are the plots of the temperatures measured by the TCs soldered on the back of the disk that registered the disk back local surface temperature histories during chilldown. Figure 6a shows a typical set of full 25 chilldown curves from a spray quenching experiment. The chilldown curves shown in Fig. 6a were obtained from the bare surface stainless-steel disk in 1-g condition. Figure 6b is a simplified version of Fig. 6a where only the medium chilldown curve for each ring is plotted in addition to the center TC25 and outer boundary TC5. A medium chilldown curve is the one with a chilldown time in the middle among those of the four curves from the same ring. At any instant, a local point on the back surface is the warmest and the corresponding point on the front surface is the coolest, therefore, chilldown at a point is considered complete only when the back temperature at that point has reached the saturated liquid temperature corresponding to the local pressure. Chilldown time is therefore defined based on the disk back surface temperatures. As seen in Fig. 6a, based on the individual TC’s distance from the center, we have divided the chilldown curves into four groups identified by red, green, yellow, and purple double-headed arrows. The red group includes the center TC and all four TCs on R1, the green group includes all eight TCs on R2 and R3, the yellow group includes all eight TCs on R4 and R5, and the purple group includes three TCs on R6. The color is also used to identify the TCs as shown in Fig. 5. For example, TCs 2, 10, 14, 22, and 25 belong to the red group. As a result, the red group is closest to the center, the green group is the next closest, the yellow group is next closest to the green group, and the purple group is the farthest. It is very clear that the closer to the center the faster the rate of chilldown. The center (TC25) is always the fastest chilldown point. As a result, all the chilldown curves are aligned in a predictable order which is that the chilldown curve from a TC that is located farther from the center is always positioned to the right side of the curve from a TC located closer to the center.

### Boiling curves

**Fig. 7:**
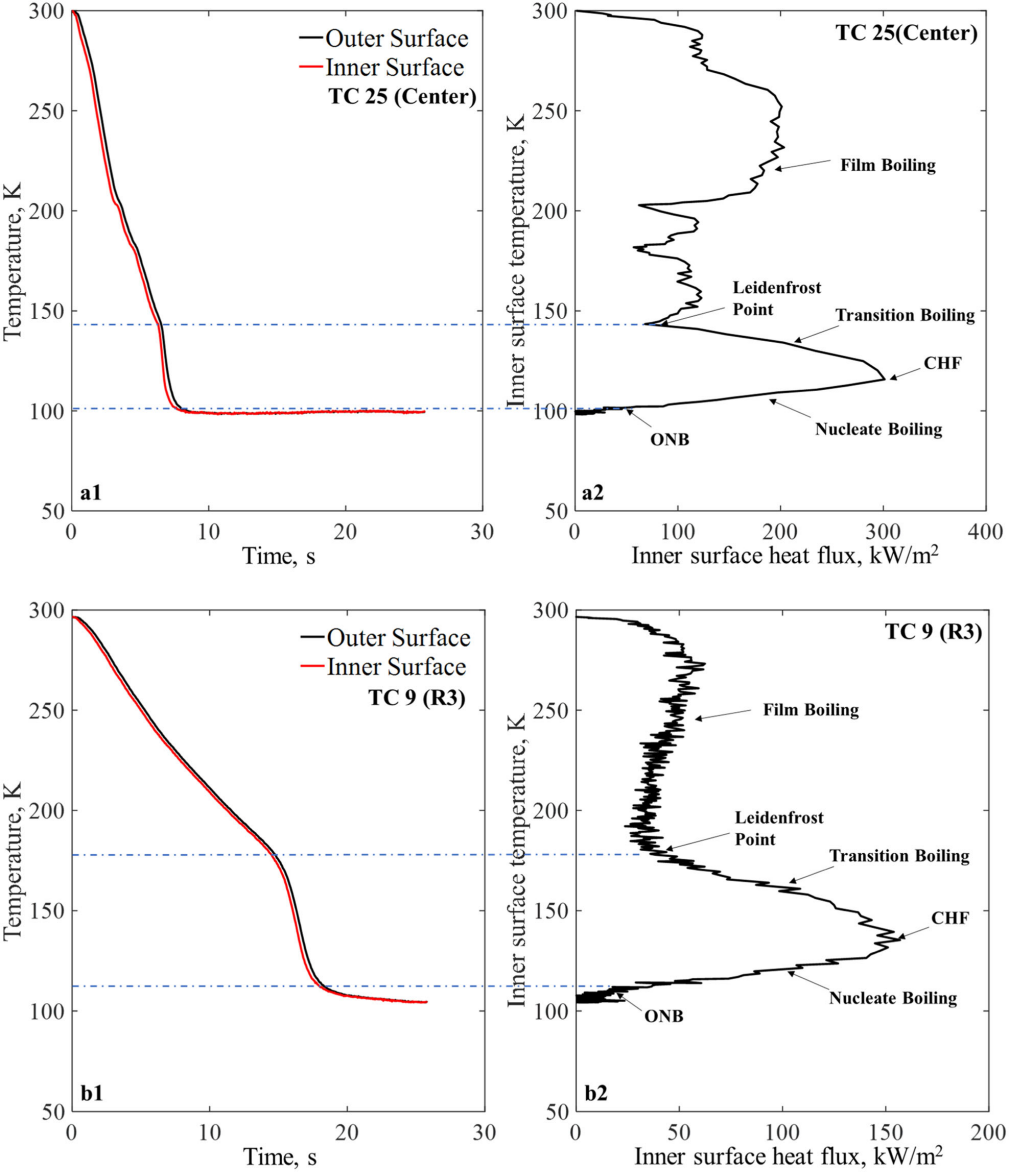
Chilldown curve and boiling curves for Case 1, 80 psig inlet pressure, and continuous flow with stainless-steel bare surface disk in micro-G. (**a**1) chilldown curve from center TC, (**a**2) boiling curve from center TC, (**b**1) chilldown curve from TC9 on Ring 3, and (**b**2) boiling curve from TC9 on Ring 3.

For every chilldown curve, there exists a corresponding unique boing curve. A boiling curve illustrates the heat transfer characteristics during chilldown by providing the inner wall transient surface heat flux as a function of inner wall surface temperature. Figure 7a2, b2 provide two sets of chilldown curve versus boiling curve plots with data obtained by the center TC25 and TC9 on Ring 3, respectively, for Case 1 using the bare surface disk. It is noted that the tube inner surface temperature and heat flux were obtained using the measured outer wall surface temperature through an inverse conduction method developed by Burgraff^22^. Readers are referred to current authors’ previous papers^17,19^ for details. The chilldown curve and corresponding boiling curve are shown in Fig. 7. Specifically, Fig. 7a, b show the corresponding boiling curves as explained in Fig. 1 where the heat transfer regimes, Leidenfrost point (LFP) and CHF point as a function of the disk wall surface temperature. According to Fig. 7a, the LFP is found just before the sharp slope change of the chilldown curve, and the almost vertical line belongs to the transition regime, CHF, and nucleate boiling regime. regimes. Comparing between Fig. 7a, b, the heat transfer rates at the center were much higher than those at the TC9 location due to higher spray mass fluxes at the disk center that resulted in much quicker chilldown time at the center.

### Disk plate chilldown thermal efficiencies

In order to measure the spray chilldow performance, we adopted a spray thermal efficiency concept that measures how much of the cooling capacity of the supplied spray cryogenic liquid is actually utilized in chilling down the disk. The chilldown efficiency as defined below represents the percent of available total quenching capacity of the liquid cryogen supplied that is actually utilized in cooling the disk (target of spray cooling) from room temperature to liquid saturation temperature of LN2 corresponding to spray chamber pressure. The spray chilldown thermal efficiency for a disk plate, $\eta _{CD}$, is therefore defined as,

2 $$\eta _{{\mathrm{CD}}} = \frac{{Q_{{\mathrm{Re}} {\mathrm{moved}}}}}{{Q_{{\mathrm{Available}}}}} \times 100\%$$

In the above, $Q_{{{{\mathrm{Removed}}}}}$ is the total thermal energy removed from the disk plate by the cooling fluid during chilldown and is defined as,

3 $$Q_{{{{\mathrm{Removed}}}}} = \left( {M_{{{{\mathrm{TARGET}}}}}} \right)c_{p,\,SS}\left( {T_{{\mathrm{initial}}} - T_{{\mathrm{final}}}} \right) + \left( {M_{{{{\mathrm{NON - TARGET}}}}}} \right)c_{p,\,SS}\left( {T_{initial} - T_{{\mathrm{NON}} - {\mathrm{TARGET}},\,{\mathrm{AVERAGE}}}} \right)$$

where $M_{{{{\mathrm{TARGET}}}}}$ is the mass of the chilldown target area that totally intercepts the spray cone and is completely cooled down by the spray fluid to the saturated liquid nitrogen temperature. It is noted that the target area is also a circular disk with a radius of r_target_ which is part of the whole disk with a radius of $r_D$ from the center. $c_{P,\,\,SS}$ is the stainless steel specific heat for the disk plate material. $T_{{\mathrm{Initial}}}$ and $T_{{\mathrm{Final}}}$ are the initial temperature when the chilldown is started and the final temperature of the target when the chilldown is completed, respectively. The end of chilldown temperature is the liquid saturation temperature corresponding to the local pressure. $M_{{{{\mathrm{NON - TARGET}}}}}$ is the non-target area that does not receive any liquid spray and is therefore equal to the total disk plate mass, $M_{{\mathrm{DISK}}\,{\mathrm{PLATE}}}$ minus that of the target area,$M_{{{{\mathrm{NON - TARGET}}}}} = M_{{{{\mathrm{DISK}}}}\,{{{\mathrm{PLATE}}}}} - M_{{{{\mathrm{TARGET}}}}}$. $T_{{{{\mathrm{NON - TARGET, AVE}}}}}$ is the estimated mass averaged temperature of the non-target area at the end of chilldown based on the conservation of thermal energy. $T_{{{{\mathrm{NON - TARGET, AVE}}}}}$ is calculated using the flowing equation,

4 $$\left( {M_{{\mathrm{NON}} - {\mathrm{TARGET}}}} \right)c_{p,\,SS}\left( {T_{{\mathrm{initial}}} - T_{{\mathrm{NON}} - {\mathrm{TARGET}},\,{\mathrm{AVERAGE}}}} \right) = Q_{{\mathrm{NON}} - {\mathrm{TARGET}}}$$

5 $$Q_{{{{\mathrm{NON - TARGET}}}}}\,\, = {\int}_0^{t_{end}} {q{\prime\prime}_{{\mathrm{Boundary}}\,\,{\mathrm{of}}\,{{{\mathrm{Target}}}}}(t)2\pi r_{{{{\mathrm{Target}}}}}t_{{\mathrm{Plate}}}dt}$$

Where $q{\prime\prime}_{{{{\mathrm{Boundary}}}}\,{{{\mathrm{of}}}}\,{{{\mathrm{Target}}}}}\left( t \right)$ is time-dependent heat flux due to heat conduction at the interface between the target and non-target areas. The non-target area is only cooled by conduction heat flow through the interface between the target and non-target areas as the non-target surface area does not receive spray droplets for cooling. $r_{{{{\mathrm{Target}}}}}$ is the radius of the target area and $t_{{{{\mathrm{plate}}}}}$ is the thickness of the disk plate. $q{\prime\prime}_{{{{\mathrm{Boundary}}}}\,{{{\mathrm{of}}}}\,{{{\mathrm{Target}}}}}\left( t \right)$ can be estimated by using the commercial software, Ansys Fluent, with the two measured chilldown curves as the boundary conditions. One chilldown curve at the outer boundary of the target area and the other at the outer boundary of the non-target area were used as the boundary conditions.

$Q_{{\mathrm{Available}}}$ is the total quenching capability supplied during the chilldown process. It is defined as,

6 $$Q_{{\mathrm{Available}}} = M_{{\mathrm{coolant}}}h_{fg}$$

Where $M_{{\mathrm{Coolant}}}$ is the total mass of coolant used and it can be estimated as,

7 $$M_{{\mathrm{Coolant}}} = {\int}_0^{t_{{\mathrm{End}}}} {\dot m\left( t \right)dt}$$

$\dot m\left( t \right)$ is the recorded time-dependent coolant mass flow rate and $t_{{\mathrm{End}}}$ is the end of chilldown time that corresponds to the time when the entire target area has reached $T_{{\mathrm{Final}}}$. Therefore, $M_{{\mathrm{Coolant}}}$ is the total mass of cryogenic coolant consumed in the entire chilldown process. $h_{fg}$ is the latent heat of vaporization per unit mass that means $Q_{{\mathrm{Available}}}$ is the available total quenching capacity.

For cases where the target area is not totally chilled down during the microgravity period, use the following for $Q_{{\Re} moved}$.

8 $$\begin{array}{l}Q_{{\mathrm{Re}} {\mathrm{moved}}} = \left( {M_{{\mathrm{TARGET}}\,{\mathrm{sub}}\,1}} \right)c_{p,\,SS}\left( {T_{{\mathrm{initial}}} - T_{{\mathrm{final}}}} \right) + \left( {M_{{\mathrm{TARGET}}\,{\mathrm{sub}}\,2}} \right)c_{p,\,SS}\left( {T_{{\mathrm{initial}}} - T_{{\mathrm{AVE}},\,{\mathrm{TARGET}}\,{\mathrm{sub}}\,2}} \right) + \\ \left( {M_{{\mathrm{NON}} - {\mathrm{TARGET}}}} \right)c_{p,\,SS}\left( {T_{{\mathrm{initial}}} - T_{{\mathrm{NON}} - {\mathrm{TARGET}},\,{\mathrm{AVERAGE}}}} \right)\end{array}$$

where*, M*_TARGET sub1_ = mass of target subregion 1 where it is completely chilled down, *M*_TARGET sub2_ = mass of target subregion 2 where it is partially chilled down, and *T*_AVE, TARGET sub2_ = average temperature of target subregion 2 at the end of chilldown, *t*_END_.

### Uncertainty of chilldown efficiency

To find the uncertainty in chilldown thermal efficiency, $\eta _{{\mathrm{CD}}}$, Eq. (2) to (8) were cast in terms of seven independent quantities. The uncertainty of the chilldown efficiency was determined by applying the individual uncertainties of the seven quantities (listed in Table 3) using the root-mean-square method. The relative uncertainties for the coated disk and bare surface disk thermal efficiencies in microgravity range between 7.40 to 7.58% and between 8.71 to 9.44%, respectively.

**Table 3:** Individual uncertainty of the independently measured quantities.

Symbol	Quantity	Measurement method	Uncertainty
*D*	Diameter of the target area	Rulers	1.6 × 10^−3^ m
*ρ*	Density of test section	NIST website	1%
*c*_*p*_	Specific heat capacity of the test section	NIST website	5%
*ΔT*	Temperature difference between the initial and the end of the test	T-type thermocouple	1.5%
*τ*	Thickness of test section	Calipers	3 × 10^5^ m
*h*_*fg*_	Latent heat of nitrogen	NIST website	5%
$\dot m_l$	Mass flow rate of liquid nitrogen	Coriolis flow meter	0.3%

Table 4 lists the estimated chilldown efficiencies using Eqs. (1)–(7) for all six cases. The mass flow rates under microgravity for all six cases are also listed. For each case, there are four entries for microgravity gravity versus terrestrial gravity. Additionally, for each gravity condition, there are comparisons between the bare surface stainless-steel disk and the Teflon coated disk. For Cases 5 and 6, chilldown efficiencies for microgravity conditions were estimated based on incomplete mass flow rates and marked by an *. The actual complete mass flow rates were not available for microgravity conditions due to some intermittent lower flow rates that were below the measurement threshold of the Coriolis flow meter due to low liquid capacity in the supply tank during later stages of the parabolic flight experiment. Therefore, these two cases are somewhat between continuous flow and intermittent flow conditions. When the liquid capacity is lower than 50% in the tank, the pressure-driven flow mechanism was not able to completely fill the entrance of the transfer pipe completely with liquid during microgravity that resulted in some mass flow rates that are lower than the flow meter measurement threshold.

**Table 4:** Chilldown efficiencies and mass flow rates for the six flight cases.

	Reduced-G			Ground-G	
Case	Teflon coated	Bare SS304	Mass flow rate, kg/s	Teflon coated	SS304
1 (micro-g) 80 psig, continuous flow	18.54%	13.38%	0.0253	23.19%	19.54%
2 (micro-g) 80 psig 40% DC, 1 s Period	28.50%	21.26%	0.0350	28.49%	21.74%
3 (micro-g) 80 psig 70% DC, 1 s Period	23.48%	13.65%	0.0239	21.17%	17.78%
4 (micro-g) 60 psig, continuous flow	28.40%	17.83%	0.0100	24.50%	18.58%
5 (micro-g) 90 psig, continuous flow	16.87%*	12.96%*	0.0264*	17.26%	14.32%
6 (micro-g) 90 psig 50% DC, 1 s Period	23.88%*	18.63%*	0.0255*	20.52%	16.76%

With the six cases investigated, the efficiencies range between 19–29% and 13–21% for coated disk and bare surface disk in microgravity, respectively. However, in terrestrial gravity, the efficiency ranges are 17–29% and 14–22% for coated disk and bare surface disk, respectively. In general, the spray chilldown (cooling) efficiencies using cryogenic fluids are significantly higher than those using water or FC-72 that normally carry efficiencies only from 5% to up to about 10%^9,10^.

### Microgravity chilldown curves

**Fig. 8:**
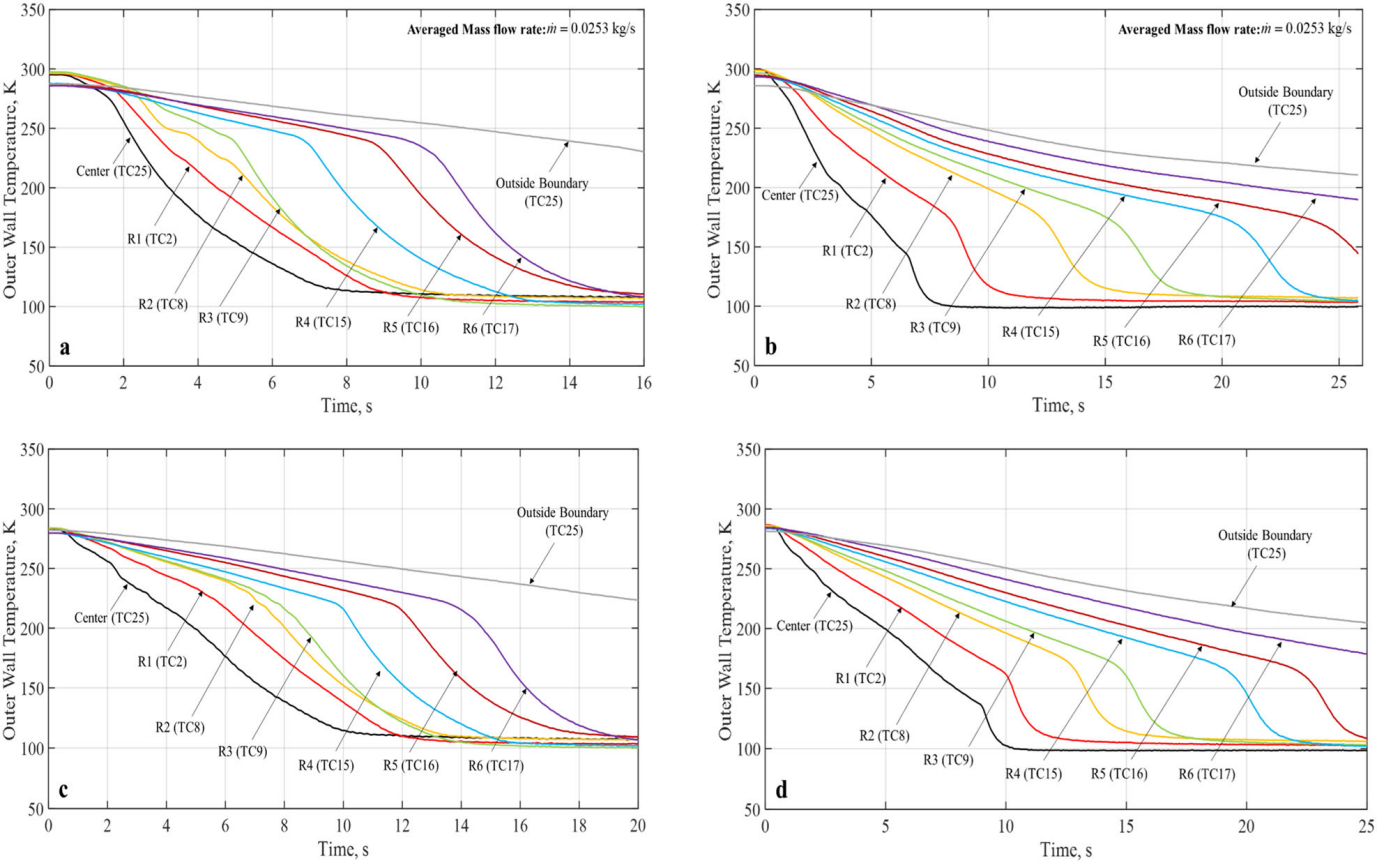
Simplified chilldown curves for 80 psig inlet pressure, continuous flow (Case 1). **a** Teflon coated disk in micro-G, **b** stainless-steel bare surface disk in micro-G, **c** Teflon coated disk in terrestrial gravity, **d** stainless-steel disk in terrestrial gravity.

Figure 8 presents the spray chilldown curves for Case 1 that has an inlet pressure of 80 psig and a continuous flow. Figure 8a, b are microgravity runs for coated and bare surface tubes, respectively. To provide a comparison, Fig. 8c, d are terrestrial counterparts for coated and bare surface tubes, respectively. The common feature for all four curves is that all curves show the typical quenching characteristics of a chilldown curve as discussed above. For both 1-g and microgravity, the coated disk was chilled down much faster than the bare surface disk. In microgravity, only the coated disk completed the chilldown within the 17-s reduced gravity time. For example, as shown in Fig. 8a, b for Case 1 up to Ring 4, the coated disk was completely chilldown in 12.8 s, while the bare surface disk took 25 s. It is worth noting that under both 1-g and microgravity, for the coated tube, the chilldown curves of the center (TC25) and Ring 1 (TC2) do not exhibit the typical sequence of starting with film boiling. In other words, these curves do not have a distinct LFP.

**Fig. 9:**
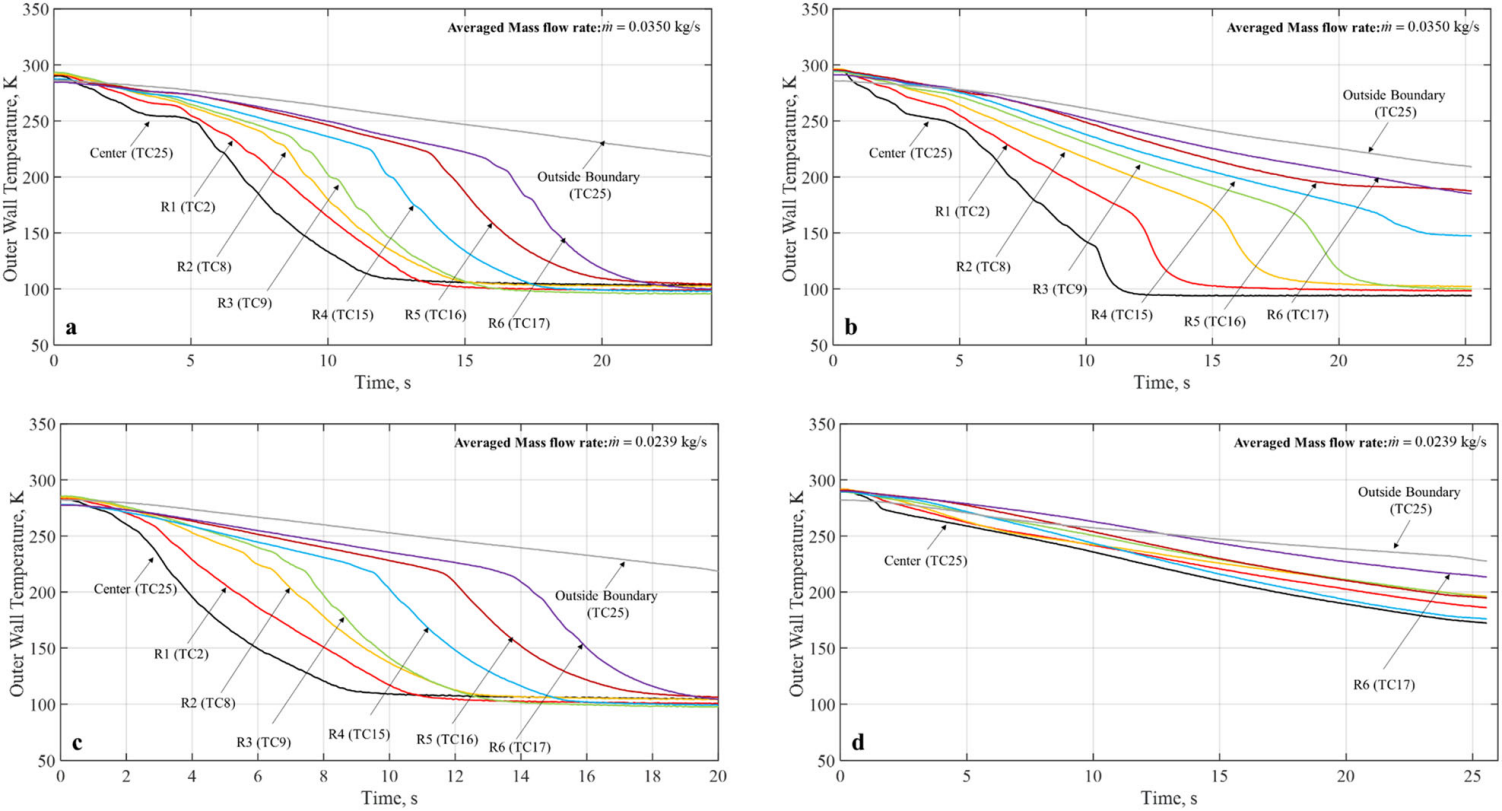
Simplified chilldown curves for micro-G, 80 psig inlet pressure. **a** 40% DC, 1 s period (Case 2) for Teflon coated disk, **b** 40% DC, 1 s period (Case 2) for stainless-steel bare surface disk, **c** 70% DC, 1 s period (Case 3) for Teflon coated disk, **d** 70% DC, 1 s period (Case 3) for stainless-steel bare surface disk.

**Fig. 10:**
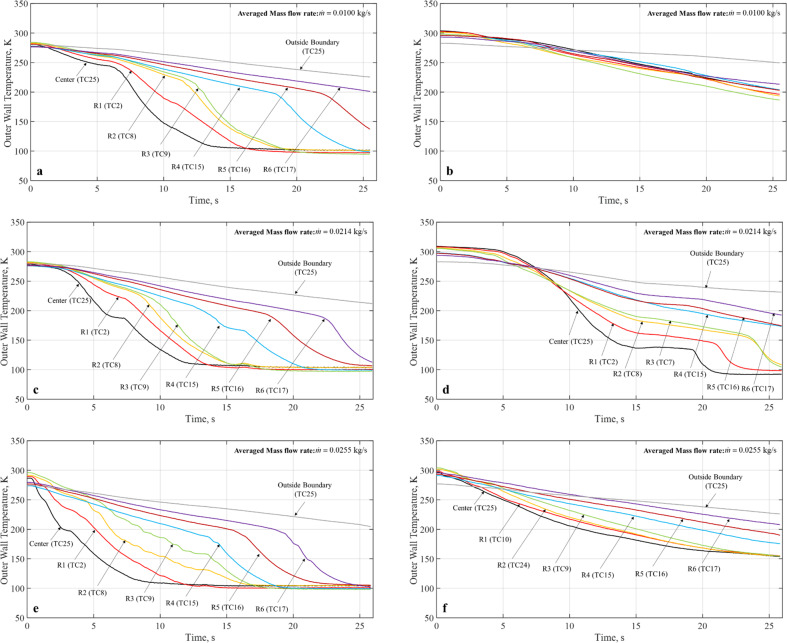
Simplified chilldown curves for micro-G. **a** 60 psig inlet pressure, continuous flow (Case 4) for Teflon coated disk, **b** 60 psig inlet pressure, continuous flow (Case 4) for stainless-steel bare surface disk, **c** 90 psig inlet pressure, continuous flow (Case 5) for Teflon coated disk, **d** 90 psig inlet pressure, continuous flow (Case 5) for stainless-steel bare surface disk, **e** 90 psig inlet pressure, 50% DC, 1 s period (Case 6) for Teflon coated disk, **f** 90 psig inlet pressure, 50% DC, 1 s period (Case 6) for stainless-steel bare surface disk.

Figures 9 and 10 show the microgravity chilldown curves for the rest of five cases. For each case, there are two plots, one for the Teflon coated disk and the other for the bare surface stainless-steel disk. It is noted that a complete chilldown up to Ring 6 within the 17–20 s of reduced gravity time was only feasible for the coated disks as the bare surface disk took much longer to reach the same conditions. For example, specifically for Case 3 (Fig. 9d), Case 4 (Fig. 10b), and Case 6 (Fig. 10f), even the center was not completely chilled down in 20 s. All the chilldown curves show similar characteristics of spray quenching cooling where the local rate of cooling heat transfer is inversely proportional to the distance between the local point and the center of the disk. In general, for all cases, the coating substantially expedited the chilldown process. For example, as shown in Fig. 9a for Case 2 up to Ring 4, the coated disk was completely chilldown in 12.8 s, while the bare surface disk took 25 s. More specifically, we will discuss quantitatively the enhancement of chilldown by the low-thermal conductivity coating below.

### Effects of gravity on disk chilldown heat transfer

Based on the spray chilldown data obtained in microgravity and terrestrial gravity, the effects of gravity on spray quenching are assessed in terms of the chilldown efficiency listed in Table 4. In general, for continuous flows, the gravity was found to enhance the chilldown efficiency for both coated and bare surface disks. The chilldown efficiencies in microgravity are 2 to 32% lower than those counterparts in terrestrial gravity for Cases 1, 4, and 5. The only exception is the coated disk in Case 4 where the efficiency is 16% higher in microgravity. However, the trend is somewhat mixed for pulse flows. For Case 2, the gravity almost made no difference. However, for Case 3, the efficiency is higher for coated tubes and lower for bare surface disks in microgravity versus in terrestrial gravity. While for Case 6, the efficiencies are higher for both coated and bare surface disks in microgravity.

To explain the above, we offer the following. For the continuous flows, the higher efficiencies in terrestrial gravity are basically due to two heat transfer enhancement mechanisms. Natural convection that is induced by gravity is the first mechanism when the film boiling is taking place on the disk surface, while the draining and thinning of the liquid film during the transition and nucleate boiling would be the second mechanism that enhances the heat transfer. However, for the pulse flows we may not have stable vapor films or liquid films for heat transfer enhancement.

### Effects of disk coating on chilldown heat transfer

In previous sections, we have already touched on some aspects of the low-thermal conductivity coating effects. Here, we would summarize those effects. Table 5 lists the percent increase in efficiency for the coated disk over the bare surface disk in both microgravity and terrestrial gravity. Consistently without any exception, the coating enhanced the heat transfer during spray chilldown as shown in Table 5 for all six cases in both microgravity and terrestrial gravity. However, for each case, the improvement in microgravity is always higher than that in terrestrial gravity that is basically due to the fact the efficiencies for bare surface disks are all lower in microgravity than those in terrestrial gravity as explained above. Therefore, there is more room for improvement. The results of coating effects thus confirm the theoretical basis for low-thermal conductivity coating given above.

**Table 5:** Improvement of chilldown efficiency by coating.

	Reduced-G	Ground-G
Case	Percent improvement	Percent improvement
1 (micro-g) 80 psig, continuous flow	38.6%	18.7%
2 (micro-g) 80 psig 40% DC, 1 s Period	34.1%	31.1%
3 (micro-g) 80 psig 70% DC, 1 s Period	72.0%	19.1%
4 (micro-g) 60 psig, continuous flow	59.3%	31.9%
5 (micro-g) 90 psig, continuous flow	30.2%	20.5%
6 (micro-g) 90 psig 50% DC, 1 s Period	28.2%	22.4%

### Effects of flow pulsing on chilldown heat transfer

Let us first compare the efficiencies for Cases 1, 2, and 3 for the flow pulsing effects as they all have the same inlet pressures of 80 psig. As can be seen from Table 4 that for Case 2 pulse flow with 40% DC and 1 s period, all four efficiencies are higher than the corresponding ones in Case 1 of continuous flow. Specifically, in microgravity, the improvements in efficiencies due to pulsing are 54 and 59% for coated and bare surface tubes, respectively. But for Case 3 with 70% DC and 1 s period, the only pulse flow improvement over the continuous flow case is 27% for the coated disk in microgravity and the other three are either about the same (bare surface in microgravity) or less efficient (coated disk and bare surface disk in terrestrial gravity). Next, between Cases 5 and 6, all four efficiencies from the pulse flow Case 6 are higher than those corresponding ones with the continuous flow Case 5. Again, in microgravity, the improvements in efficiencies due to pulsing are 42 and 44% for coated and bare surface tubes, respectively. We may conclude that pulse flows can improve the chilldown efficiency, but only with lower duty cycles.

### Combined effects of disk coating and flow pulsing on chilldown heat transfer in microgravity

First, let us compare the coated disk of Case 2 and the bare surface disk of Case 1 in microgravity. We found that the improvement due to both coatings, and 40% DC and 1 s period pulse flow is 113% for a common inlet pressure of 80 psig. The other comparison is between the coated disk of Case 6 and the bare surface disk of Case 5 in microgravity. The improvement due to both coating, and 50% DC and 1 s period pulse flow is 84% for a common inlet pressure of 90 psig.

### Effects of inlet pressure on chilldown heat transfer

Cases 1, 4, and 5 provide information for assessing the inlet pressure effects. First, we need to stress that the mass flow rate is actually proportional to the inlet pressure as the flow is driven by the pressure difference between the inlet pressure and the pressure at the outlet. Since the outlet pressure is relatively constant that makes the mass flow rate to be totally dependent on the inlet pressure. As a result, it is the case that the higher the inlet pressure, the higher the mass flow rate. As explained above, the chilldown efficiency is inversely proportional to the mass flow rate that leads to the outcome that Case 4 should possess the highest efficiency, Case 1 is in the middle, and Case 5 is the least efficient for each of the four categories (coated disk in microgravity, bare surface disk in microgravity, coated disk in 1-g, and bare surface disk in 1-g). It turned out that only the bare surface disk in 1-g did not follow the predicted trend, where Case 1 has the highest efficiency instead of Case 4 and Case 5 is still the lowest.

## Discussion

The most important finding in the current research is that the bulk of the spray quenching enhancement and the corresponding spray cooling thermal efficiency improvement is largely due to the low-thermal conductivity thin-film coating, especially in microgravity. As mentioned above, the poor heat transfer film boiling regime occupies the major portion of the chilldown time for the bare surface disk without coating that also translates into low quenching thermal efficiency. The low-thermal conductivity coating can facilitate a quick drop of the disk surface temperature that expedites the approach to the LFP on the disk surface and the switch over from the film boiling regime to the transition boiling regime, thus drastically shortens the film boiling time and increases the rates of heat transfer. The conduction theory also predicts that the thicker the coating, the quicker the surface reaches the LFP. However, once the quenching process enters the high heat transfer regimes of transition boiling and nucleates boiling, the coating becomes an insulator that results in lower heat transfer rates as compared to those of bare surface disks. As a result, after reaching the LFP it is required that the thickness of the coating material be as thin as possible to expedite the disk cooling process. Based on the two scenarios, there should be an optimal coating thickness such that it is not too thick to drastically reduce the conduction of heat from the disk to the cooling fluid, but also still thick enough to quickly drop the surface temperature to the LFP. Since we only had one flight, we were not able to test different coating thicknesses.

## Supplementary information

Reporting summary checklist

## Data Availability

The authors declare that the data supporting the findings of this study are available within the paper.
